# A Rare Case Report on Postoperative Rehabilitation in Hirschsprung Disease

**DOI:** 10.7759/cureus.54044

**Published:** 2024-02-12

**Authors:** Shifa S Sheikh, H V Sharath, Nikita H Seth

**Affiliations:** 1 Department of Paediatric Physiotherapy, Ravi Nair Physiotherapy College, Datta Meghe Institute of Higher Education & Research (Deemed to be University), Wardha, IND; 2 Department of Neurophysiotherapy, Ravi Nair Physiotherapy College, Datta Meghe Institute of Higher Education & Research (Deemed to be University), Wardha, IND

**Keywords:** pediatric rehabilitation, hirschsprung disease, paediatric hirschsprung disease, paediatric physiotherapy, absence of ganglion cells

## Abstract

Hirschsprung disease (HD) is characterized by the absence of ganglion cells in the myenteric and submucosal plexuses of the hindgut. Here, we report a case of an eight-year-old male child who had abdominal distension with a history of repetitive gas passage and a complaint of stool passage. In February 2023, the patient was diagnosed with Hirshsprung disease, for which a left-side colostomy was done. In November 2023, he underwent Hirshsprung stage 2 repair. He was operated on the 17th of December 2023 under general anaesthesia colostomy mobilization. Physiotherapy commencement and evaluation were started on the 18th of December 2023. After the colostomy procedure, the incision weakened the abdominal and lower limb muscles, while bowel obstruction and discomfort further impeded the patient's ability to perform daily activities. Physical examination revealed increased work of breathing, reduced range of motion of the bilateral hip joint, reduced muscle strength of lower limb musculature, reduced abdominal muscle strength, difficulty in walking and waddling type of gait. Physiotherapy goals were set based on the problem list. The patient showed improvement in the two weeks of physiotherapy commencement, followed by improvement in functional ability. The comprehensive care provided during the rehabilitation phase aimed at addressing the specific needs arising from the surgical intervention, promoting optimal bowel function, improving ranges and strength and ensuring overall well-being.

## Introduction

Hirschsprung disease (HD) is characterized by the absence of ganglion cells in the myenteric and submucosal plexuses of the hindgut. It is an enteric nervous system developmental condition. HD causes a lack of peristalsis in the impacted colon, and a functional intestinal blockage develops [1]. The incidence rate of HD was one in 5,400-7,200 live births, with a male predominance of 4:1 [1,2]. The diagnostic process for HD is essential for confirming the condition, and procedures such as rectal biopsy play a pivotal role as a primary diagnostic tool. A delayed diagnosis of HD could be associated with a poor prognosis [2]. An early halt in the movement of vagal neural crest cells within the hindgut, typically occurring between the fifth and 12th weeks of gestation. This interruption prevents the formation of the enteric nervous system (ENS) and is classified as a neurocristopathy [3].

Newborns with intestinal obstruction present with delayed passage of meconium, feeding intolerance, swollen abdomen, often relieved through rectal stimulation or enemas, and neonatal enterocolitis. In some cases, individuals are diagnosed later in infancy or adulthood with severe constipation, persistent abdominal swelling, vomiting, dependence on rectal therapy and failure to grow adequately [4]. Congenital anomalies associated with this condition may encompass abnormalities in the cardiac, gastrointestinal, limb, genitourinary and the central nervous and peripheral neuronal systems. Other causes of intestinal obstructions are meconium ileus due to cystic fibrosis, colonic atresia, lower ileal atresia, long-term intestinal pseudo-obstruction syndrome and intestinal malrotation [5].

Rectal biopsy aids in recognizing the lack of ganglion cells within the affected colon segment. Additional diagnostic measures include manometry, which assesses anal sphincter pressure and relaxation, colonic transit studies for tracking marker movement in the colon, genetic testing to assess specific gene mutations, ultrasound imaging and anorectal ultrasonography, which gives a detailed imaging of the size and shape of the colon, anus and rectum [6]. HD is categorized into short-segment HD when the aganglionic segment is confined to the rectum and sigmoid colon. On the other hand, long-segment HD extends beyond the sigmoid colon and encompasses more extensive portions of the colon [7].

A delayed diagnosis of HD could be associated with an unfavourable prognosis [8]. The surgical care of HD attempts to preserve normal sphincter function while excising the aganglionic intestine and reconstructing the digestive tract by bringing the innervated bowel to the anus. The following surgical techniques are used: Swenson, Duhamel, Soave and colostomy [9]. When it is anatomically difficult for faecal excretion to pass through the anal canal, a colostomy is a surgical treatment intended to divert intestinal transit. This operation is done surgically by making an incision in the colon wall and posteriorly exteriorizing it in the abdominal wall, which permits the expulsion of gasses and waste [10]. The complication rate of colostomy in HD was 74.5%. A higher incidence of prolapse occurs in HD due to the weaker abdominal wall, increased abdominal pressure, higher mobile bowel mesentery and large openings during stroma formation. The quality of life is significantly impacted by having a colostomy [11,12]. To minimize postoperative complications and prevent failure following abdominal surgery, early physiotherapy intervention plays an essential role. Recent research indicates that engaging in just 10 minutes of dynamic exercise at a moderate-to-high intensity has a positive impact on power performance in children [13].

Enhancing core muscular endurance contributes to spinal stabilization and offers protection against the occurrence of lower back pain. Nevertheless, the implementation of effective core conditioning programs in accessible environments is crucial to improving trunk and core muscular endurance, particularly in children [14].

## Case presentation

Patient information

As narrated by the mother, her eight-year-old male child was apparently alright at the time of birth; the child had abdominal distension with a history of repetitive gas passage and complaint of stool passage every third day. In February 2023, they visited Acharya Vinoba Bhave Rural Hospital (AVBRH) and the child was diagnosed with HD, for which a left-side colostomy was done in 2023. The mother gave no history of complications during pregnancy. The child was born full term through natural vaginal delivery. The child was admitted to the neonate intensive care unit (NICU) on the third day of life due to repetitive vomiting and abdominal distension. The child had jaundice on the eighth day of life. In November 2023, the child was called for HD stage 2 repair. After a thorough examination, the child was operated on the 17th of December 2023 under general anaesthesia, and colostomy mobilization was done. Following colostomy mobilization, intra-abdominal left testis was noted near the deep ring; hence, orchidopexy of the left side was also done in the same sitting. The timeline of the events is given in Table 1.

**Table 1:** Sequence of events NICU: neonate intensive care unit

Occurrence of events	Date of events
NICU admission for repetitive vomiting and abdominal distension	Third day of life
Jaundice	Eight day of life
Diagnosis of Hirshsprung disease	February 2023
Left-side colostomy	February 2023
Stage 2 repair of Hirshsprung disease	November 2023
Colostomy mobilization and orchidopexy	17th of December 2023
Physiotherapy evaluation and commencement	18th of December 2023

Clinical findings

A well-informed consent was taken before the examination. The child was conscious, cooperative and aware of the surroundings. The child was ectomorphic in built. On observation, the child was seen in a supine lying position with his upper limbs supported by the side of the body and the hip and knee flexed to 90 degrees, with the ankle supported to the bed. His chest was bilaterally symmetrical, and the breathing pattern was abdominothoracic. The external appliances applied were a Ryles tube, Foley catheter, intravenous line, drainage tube and rectal tube that was intact. On examination, the child was afebrile with a pulse rate of 132 beats per minute, respiratory rate of 30 breaths per minute, oxygen saturation of 100 percentage and blood pressure of 110/60 mmhg. Pain on the visual analogue scale was 7/10 on rest and activity. On physical examination, the tone was normal. The range of motion of the bilateral hip joint was reduced, and the child complained of low back pain. Manual muscle strength of the bilateral hip flexors, adductors and abductors was reduced. The strength of the upper, lower and oblique abdominal muscles was reduced. All deep tendon reflexes were normal. The patient had difficulty in walking, and his gait was of waddling type.

Diagnostic investigation

**Figure 1:**
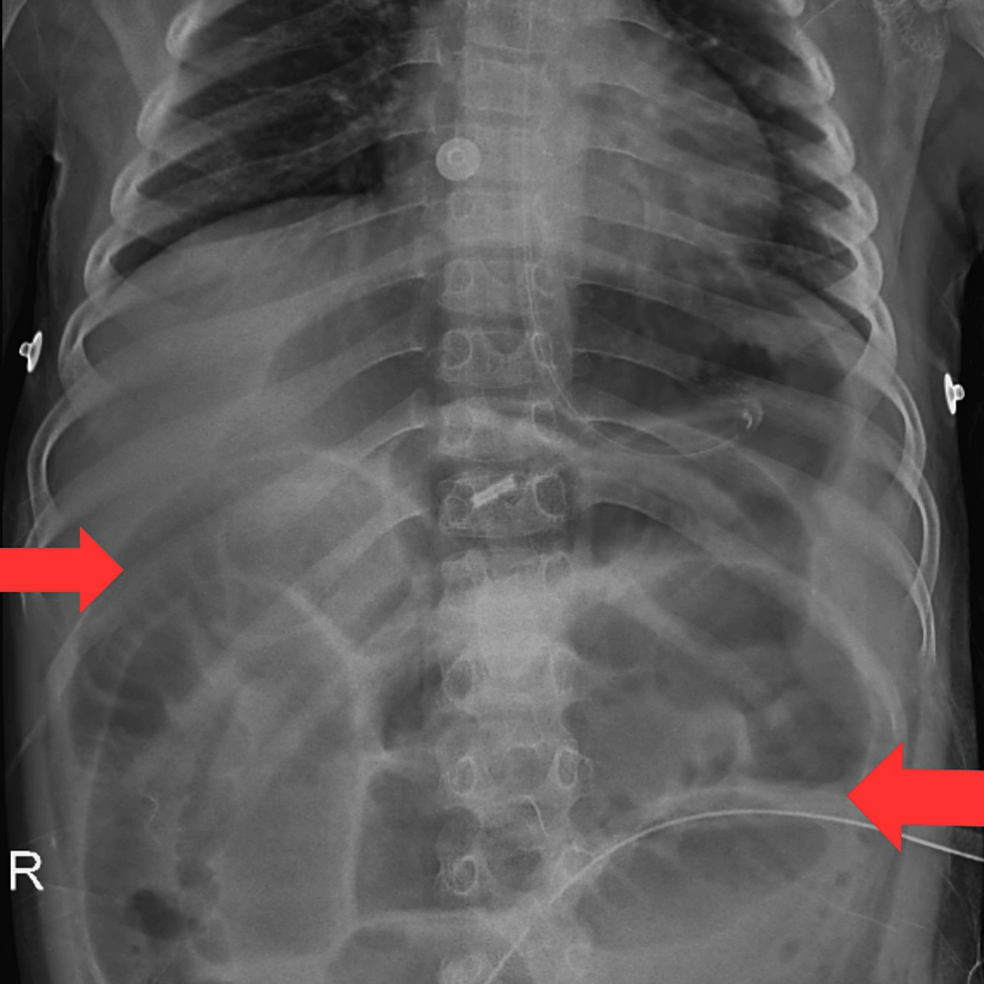
Abdominal X-ray showing multiple colon loops with haustration (indicated by the red arrow) Multiple dilated bowl loops with haustrations, suggestive of a toxic megacolon with colostomy seen over the right side.

**Figure 2:**
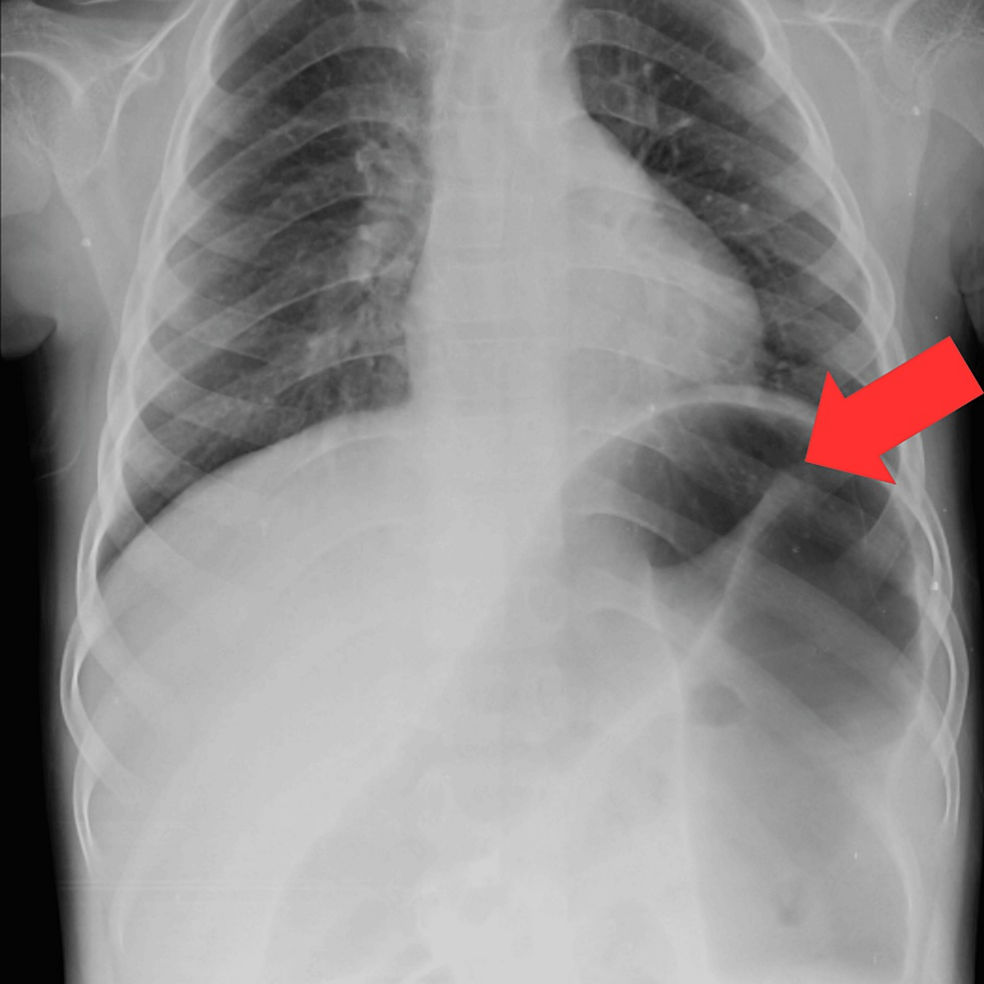
X-ray of the abdomen with the red arrow indicating a single loop of the dilated proximal colon There are fewer dilated loops observed in comparison to Figure 1, indicating a potential improvement and resolution of the condition.

An excised specimen of the descending colon and rectum was sent for a histopathology report. The cut section of the proximal part showed a partial loss of the mucosal fold. The section from the distal margin and rectum showed aganglionic cells. Sections from the area with normal mucosal folds were aganglionic on histopathology. Histopathological features suggest clinical diagnosis of HD. On abdominal X-ray, a distended small bowel and proximal colon, with absence of rectal gas, having multiple large bowel loops with haustration are shown in Figure 1. Figure 2 shows a single-loop dilated proximal colon with the aganglionic cone narrowing towards the distal gut.

Physiotherapy intervention

The goals along with the physiotherapy intervention planned and prescription of treatment are shown in Table 2.

**Table 2:** Goals and physiotherapy interventions planned and prescription of treatments ROM: range of motion; b/l: bilateral

Goals	Physiotherapy intervention	Prescription
To educate family and child about the uses of physiotherapy and its effects on daily activity	The child was educated about his condition and ways to improve his condition and daily activity through different exercises.	Before every physiotherapy session, the child was encouraged to the exercise regime.
To reduce the excess work of breathing	Pursed lip breathing and diaphragmatic breathing exercises, the diaphragm is supported during breathing, reducing the diaphragmatic workload (Figure 3)	10 repetitions x 1 set progressed to 10 repetitions x 1 set from day 1 to day 21
To improve chest expansion	Thoracic expansion exercise with upper limb mobility exercise, segmental breathing exercise focusing on lower lobe expansion	10 repetitions x 1 set progressed to 10 repetitions x 1 set from day 1 to day 21
To improve effective chest clearance	Huffing and coughing techniques	5 repetitions x 1 set from day 1 to day 7
To relieve low back ache and gluteal pain	Positioning in supine lying with a pillow under the patient’s thigh, static back exercise	Whenever the patient was lying in bed from day 4 to day 14
To reduce muscle spasm	Cryotherapy, gentle stroking	Ice pack application to the affected part for 30 seconds with a hold of 30 seconds for 5 minutes from day 1 to day 5
To improve digestive movement	A brisk walk after meals, quadruped position	Walk of 10-15 minutes, core strengthening exercises and quadruped position for 5 repetitions of 1 set from day 7 to day 28
To improve abdominal muscle strength	Initially with static abdominal strengthening followed by core strengthening exercises, lower trunk rotation (Straight leg raise (Figure 5), static curl-up (Figure 4), lateral plank, quadruped position with its progressions)	10 repetitions of 1 set From day 7 to day 28
To improve joint ROM	Initially active assisted exercises followed by active exercises of b/l upper and lower limbs	10 repetitions of 1 set, progressed to 15 repetitions of 1 set from day 1 to day 28
To strengthen the upper and lower limb muscle ROM	Strengthening exercises of b/l upper and lower limb muscle groups progressed with half kg weight	10 repetitions of 1 set, progressed to 15 repetitions of 1 set from day 1 to day 28
To restore functional independence	Functional training, including bed mobility exercises, functional re-education	Rolling from side to side (5 cycles progressed to 10 cycles), bridging exercises (5 repetitions with 5-second hold x 1 set), standing while playing with toys (5 minutes progressed to 10 minutes) starting from day 6 to 14^th^ days, standing on one leg (5 repetition with 5-second hold 1 set), squatting exercise with support progressed without support (5 repetition x 1 set progressed to 10 repetition) from day 14 to day 28^th^
Improve affected gait pattern	Gait training in parallel bar	Daily for 5 minutes from day 3 to day 14

**Figure 3:**
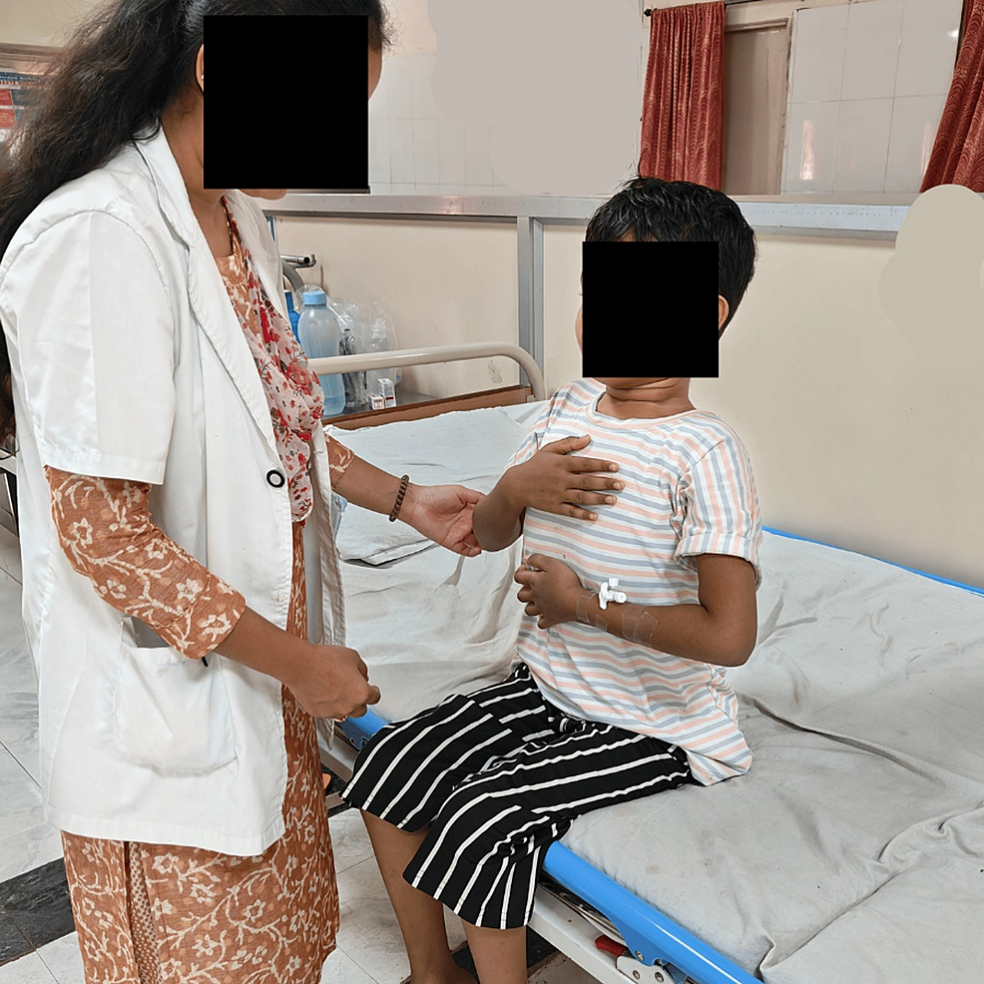
Patient doing a diaphragmatic breathing exercises

**Figure 4:**
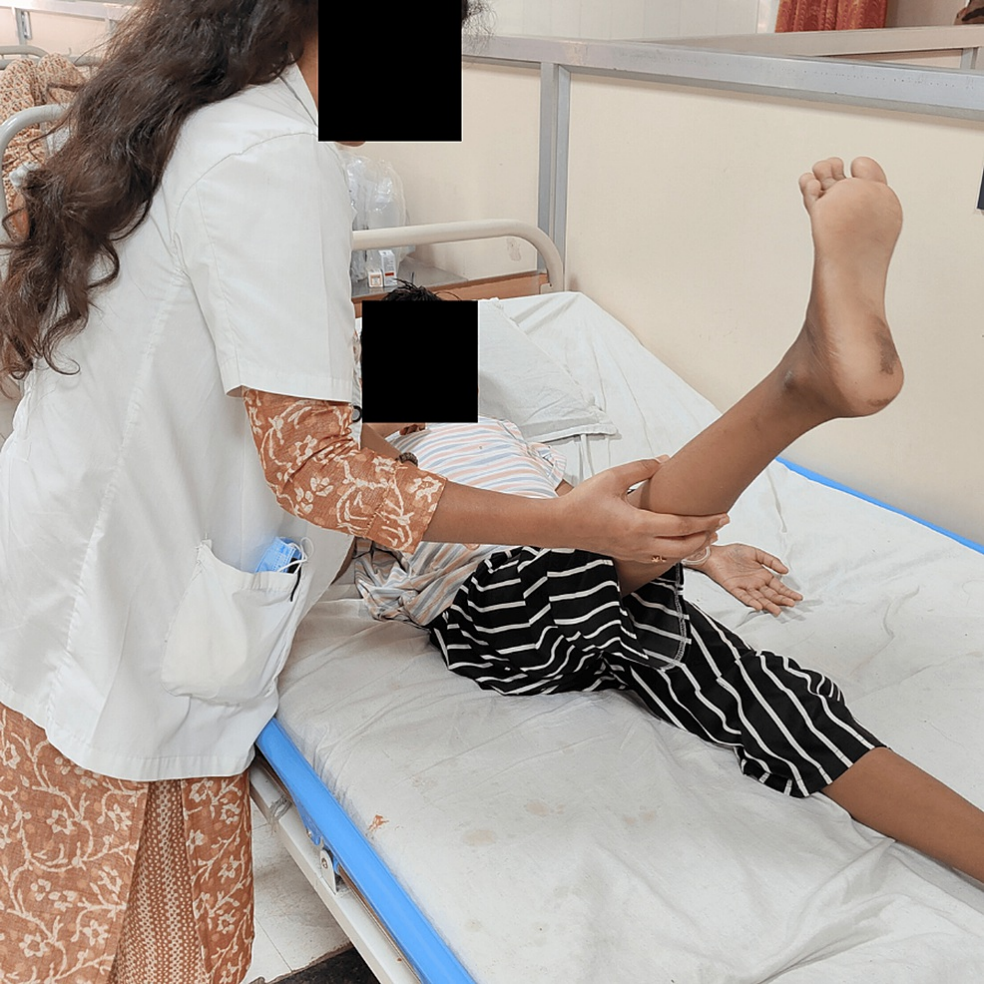
Patient doing a straight leg raise with assistance

**Figure 5:**
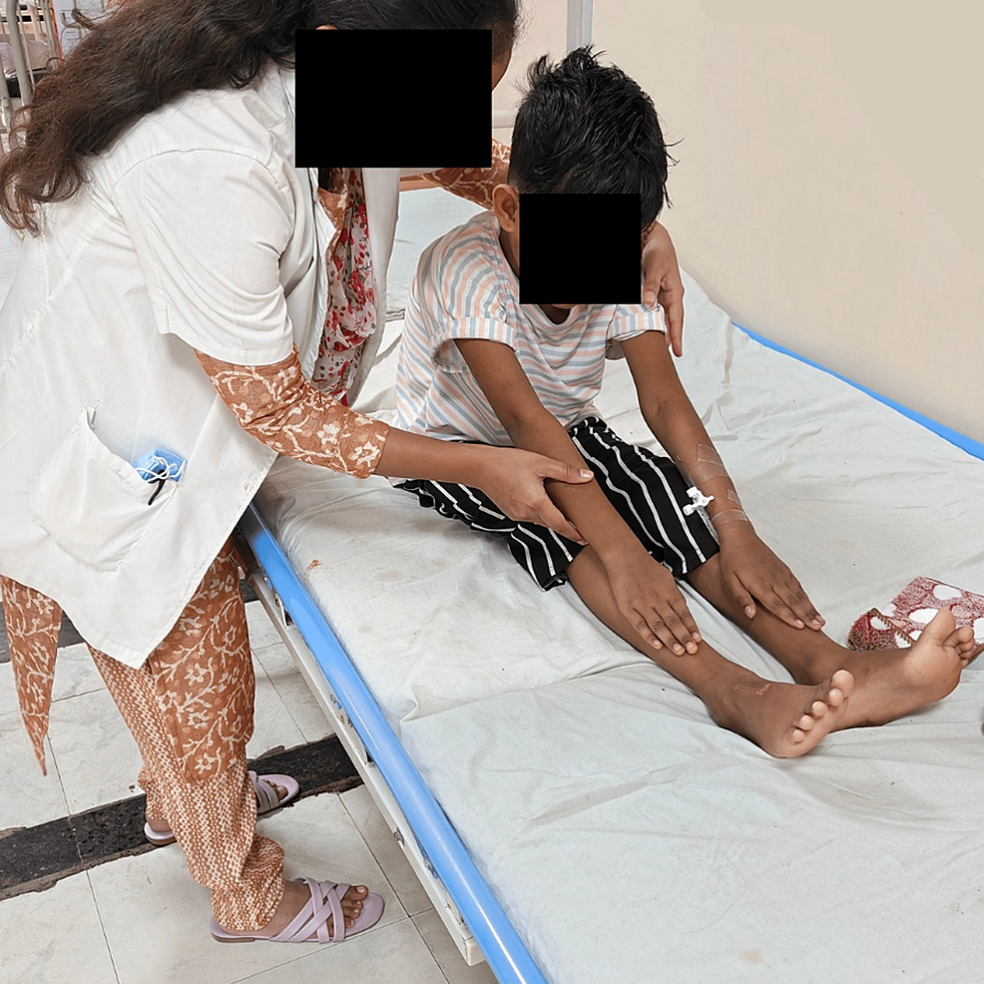
Patient doing abdominal strengthening exercises with assistance

Figures 3-5 show the patient performing a diaphragmatic breathing exercise, static curl-ups and straight leg raise, respectively. 

Follow-up and outcome measures

This case report presents the physiotherapy evaluation and rehabilitation of an eight-year-old with HD for which he was medically managed with colostomy following the removal of a stoma bag and commencement of HD repair physiotherapy intervention. The physiotherapy plan of care and treatment was in collaboration with the patient and caregivers (parents). The patient showed improvement in the two weeks of physiotherapy commencement followed by improvement in functional ability. The pain of the patient was reduced to 2/10 on the visual analogue scale. The patient initiated tasks of daily living, like playing activities involving the lower limbs and bending without fatigue. The range of motion of the hip joint is given in Table 3. The strength of the lower limb muscle group improved, as shown in Table 4. The abdominal muscle strength of the child improved, as shown in Table 5. The gait pattern of a child was significantly enhanced. Table 6 illustrates the outcome measures taken before and after treatment.

**Table 3:** Range of motion of the affected joint

Range of motion	Pre-treatment	Post-treatment
Hip flexion	0-90^o^	0-110^ o^
Hip extension	0-15^ o^	0-25^ o^
Hip abduction	0-26^ o^	0-38^ o^
Hip adduction	0-18^ o^	0-26^ o^

**Table 4:** Manual muscle testing grades pre- and post-intervention

Muscle group	Pre-treatment	Post-treatment
Hip flexors	2/5	4/5
Hip extensors	2/5	4/5
Hip adductors	3/5	5/5
Knee flexors	3/5	5/5
Knee extensors	3/5	5/5

**Table 5:** Manual muscle testing grades for the abdominal muscles

Muscle group	Pre-treatment	Post-treatment
Oblique trunk flexors	Fair (5) Grade	Good (8) Grade
Lower abdominal trunk flexors	Fair (5) Grade	Good (8) Grade
Upper abdominal muscle	Fair (5) Grade	Good (8) Grade

**Table 6:** Outcome measures

Scale	Pre-treatment score	Post-treatment score
Functional mobility scale	Level 2	Level 1
Wee Functional independence measure	54/126	108/126
Manual ability classification system	Level 2	Level 1

## Discussion

The major finding of this rare case report on postoperative rehabilitation in HD underscores the significance of tailored rehabilitation protocols in optimizing outcomes for individuals undergoing surgical intervention for HD. Through meticulous postoperative management, including early mobilization, specialized dietary adjustments and meticulous bowel management strategies, the patient achieved notable improvements in bowel function and quality of life. This highlights the importance of comprehensive multidisciplinary care in addressing the complex needs of individuals with HD postoperatively, offering valuable insights for guiding future rehabilitation strategies and enhancing long-term outcomes in this patient population.

Postoperative rehabilitation in HD is a critical aspect of care aimed at optimizing the health and well-being of patients following surgical intervention. HD, a congenital disorder affecting the large intestine, necessitates surgical removal of the aganglionic segment. This case report focuses on detailing the rehabilitation strategies employed in a specific patient post-surgery. Understanding the challenges associated with HD and recognizing the significance of comprehensive rehabilitation is essential for improving patient outcomes.

Postoperative challenges in HD often include bowel dysfunction, nutritional deficiencies and potential psychological impacts. A multifaceted rehabilitation approach is crucial for addressing these issues. Physiotherapy plays a pivotal role in enhancing bowel function and promoting abdominal muscle strength. Tailored exercises, considering the patient's age and surgical specifics, are implemented to improve mobility and reduce postoperative complications. Nutritional support is another integral component, focusing on addressing the unique dietary needs of individuals with HD to ensure optimal growth and development. Moreover, psychological support is vital for both the patient and their family, acknowledging the potential emotional toll of the disease and surgery.

The success of postoperative rehabilitation is assessed through careful follow-up and monitoring of outcomes. Regular check-ups allow healthcare professionals to evaluate the effectiveness of the rehabilitation plan, make adjustments as needed and address any emerging issues promptly. Positive outcomes may include improvements in bowel function, weight gain and overall quality of life for the patient. By highlighting the tangible benefits of the rehabilitation process, this case report contributes valuable insights to the broader understanding of postoperative care in HD. Respiratory training improved maximal exercise capacity and functional performance in children with congenital diaphragmatic hernia. By actively engaging the diaphragm through diaphragmatic breathing, individuals promote a more efficient and controlled breathing pattern. Chest resistance and expansion breathing ensures the full expansion and contraction of the lungs, facilitates better oxygenation and strengthens respiratory muscles. This process helps maintain optimal respiratory function in postoperative recovery and respiratory rehabilitation [15,16].

Santamaria et al. demonstrated that including hip musculature strengthening exercises in a lower-specific exercise regimen significantly improves pain and disability reduction in patients with low back pain (LBP) without endangering their health [17]. Queiroz et al. suggested that the quadruped position is effective in muscle activation. It engages core muscles, involves isometric contractions, promotes dynamic stability, integrates with limb movements, encourages functional alignment of the spine and is generally low-impact. Variations in pelvic and trunk position change the activation pattern of abdominal and gluteal muscles [18]. Brisk walking and physical activity after a meal can alleviate constipation by promoting bowel movement through stimulation of gastrointestinal muscle, improving defecation pattern, reducing transit time of food and reducing stress [19]. Functional training for patients involves a holistic approach to help individuals regain strength and mobility to overcome postoperative complications. Mobility and flexibility exercises, posture training, progressive strength training and aerobic training enhance functional capacity [20].

In conclusion, postoperative rehabilitation in HD is a multifaceted endeavour encompassing physiotherapy, nutritional support and psychological care. This case report sheds light on the practical implementation of rehabilitation strategies, emphasizing the importance of a comprehensive and individualized approach. By sharing the experiences and outcomes of this specific case, healthcare professionals can refine their understanding of effective rehabilitation protocols, ultimately improving the long-term prognosis and quality of life for individuals with HD.

## Conclusions

This rare case report sheds light on the significance of personalized postoperative rehabilitation strategies in enhancing outcomes for patients with HD. By implementing a tailored rehabilitation protocol involving early mobilization, specialized dietary modifications and meticulous bowel management techniques, significant improvements in bowel function and overall quality of life were observed in the patient. The comprehensive care provided during the rehabilitation phase aimed at addressing the specific needs arising from the surgical intervention, promoting optimal bowel function, improving ranges and strength and ensuring overall well-being. Moving forward, further research and clinical efforts are warranted to refine and optimize rehabilitation protocols, ultimately improving long-term outcomes and quality of life for individuals living with this condition. A child with HD showed overall improvement in motor skills, suggesting that the use of manual therapy and positioning for bowel movement activity were effective in his treatment. The ability to participate actively has contributed to his full participation in society. As this case report highlights the positive impact of physiotherapy on HD, further studies are required to explore the potential of electrotherapy modalities using different modes in the management of HD along with a refined rehabilitation protocol. These findings underscore the importance of comprehensive multidisciplinary care in addressing the complex needs of individuals with HD following surgery.

## References

[1] Negash S, Getachew H, Tamirat D, Mammo TN (2022). Hirschsprung disease managed with one-stage transanal endorectal pullthrough in a low-resource setting without frozen section. BMC Surg.

[2] (2024). Orphanet: hirschsprung disease. https://www.orpha.net/consor/cgi-bin/OC_Exp.php.

[3] Amiel J, Sproat-Emison E, Garcia-Barcelo M (2008). Hirschsprung disease, associated syndromes and genetics: a review. J Med Genet.

[4] Teeraratkul S (2003). Transanal one-stage endorectal pull-through for Hirschsprung's disease in infants and children. J Pediatr Surg.

[5] Heuckeroth RO (2018). Hirschsprung disease - integrating basic science and clinical medicine to improve outcomes. Nat Rev Gastroenterol Hepatol.

[6] Ambartsumyan L, Smith C, Kapur RP (2020). Diagnosis of Hirschsprung disease. Pediatr Dev Pathol.

[7] Gorbatyuk OM (2022). Current approaches to diagnosis and treatment of Hirschsprung disease in newborns and infants (literature review and first-hand experience). Wiad Lek.

[8] Tan YW, Chacon CS, Geoghegan N, Saxena A, Clarke S, Haddad M, Choudhry M (2022). Late diagnosis of hirschsprung’s disease: definition and implication on core outcomes. Eur J Pediatr Surg.

[9] Dasgupta R, Langer JC (2004). Hirschsprung disease. Curr Probl Surg.

[10] Campos K, Bot LHB, Petroianu A, Rebelo PA, Souza AAC, Panhoca I (2017). The impact of colostomy on the patient’s life. J Coloproctol.

[11] Ekenze SO, Agugua-Obianyo NE, Amah CC (2007). Colostomy for large bowel anomalies in children: a case controlled study. Int J Surg.

[12] Boutry E, Bertrand MM, Ripoche J, Alonso S, Bastide S, Prudhomme M (2021). Quality of life in colostomy patients practicing colonic irrigation: an observational study. J Visc Surg.

[13] Faigenbaum AD, Bellucci M, Bernieri A, Bakker B, Hoorens K (2005). Acute effects of different warm-up protocols on fitness performance in children. J Strength Cond Res.

[14] Hsu SL, Oda H, Shirahata S, Watanabe M, Sasaki M (2018). Effects of core strength training on core stability. J Phys Ther Sci.

[15] Azab AR, Abdelbasset WK, Alrawaili SM, Elsayed AE, Hajelbashir MI, Kamel FH, Basha MA (2022). Effect of chest resistance and expansion exercises on respiratory muscle strength, lung function, and thoracic excursion in children with a post-operative congenital diaphragmatic hernia. Int J Environ Res Public Health.

[16] Moawd SA, Azab AR, Ibrahim ZM, Verma A, Abdelbasset WK (2020). Impacts of respiratory muscle training on respiratory functions, maximal exercise capacity, functional performance, and quality of life in school-aged children with postoperative congenital diaphragmatic hernia. Dis Markers.

[17] Santamaría G, Rodríguez I, Rodríguez-Pérez V, Cobreros-Mielgo R, Lantarón-Caeiro E, Seco-Casares M, Fernández-Lázaro D (2023). Effect of hip muscle strengthening exercises on pain and disability in patients with non-specific low back pain—a systematic review. Sports (Basel).

[18] Queiroz BC, Cagliari MF, Amorim CF, Sacco IC (2010). Muscle activation during four Pilates core stability exercises in quadruped position. Arch Phys Med Rehabil.

[19] De Schryver AM, Keulemans YC, Peters HP, Akkermans LM, Smout AJ, De Vries WR, van Berge-Henegouwen GP (2005). Effects of regular physical activity on defecation pattern in middle-aged patients complaining of chronic constipation. Scand J Gastroenterol.

[20] van Rooijen S, Carli F, Dalton S (2019). Multimodal prehabilitation in colorectal cancer patients to improve functional capacity and reduce postoperative complications: the first international randomized controlled trial for multimodal prehabilitation. BMC Cancer.

